# IL-31, itch and hematological malignancies

**DOI:** 10.1186/s12948-021-00148-7

**Published:** 2021-06-12

**Authors:** Eleonora Di Salvo, Alessandro Allegra, Marco Casciaro, Sebastiano Gangemi

**Affiliations:** 1 Department of Veterinary Sciences, University of Messina, 98168 Messina, Italy; 2 Division of Hematology, Department of Human Pathology in Adulthood and Childhood “Gaetano Barresi”, University of Messina, 98125 Messina, Italy; 3 School and Operative Unit of Allergy and Clinical Immunology, Department of Clinical and Experimental Medicine, University of Messina, 98125 Messina, Italy

**Keywords:** IL-31, IL-33, Itch, Pruritus, Malignancies, Cancer, Cytokines, Skin

## Abstract

Pruritus is one of the most common symptoms experienced by neoplastic patients. The pathogenesis of neoplastic itch is complex and multifactorial and could be due to an unbalanced production of humoral mediators by altered immune effector cells. IL-31 is a pro-inflammatory cytokine produced by CD4 + T helper cells. The aim of this review was to evaluate the role of this Th2 cytokine and its receptor IL-31RA, in the onset of neoplastic pruritus. We analysed scientific literature looking for the most relevant original articles linking IL-31to itch in oncologic diseases. Interleukin-31 seems to be a main itch mediator in several hematologic disease such as Cutaneous T cells lymphomas. In these patients IL-31 was positively linked to itch level, and IL-31 matched with disease stage. IL-31 seems to play an important role in the signalling pathway involved in pruritus, but it is also suggested to play a proinflammatory and immunomodulatory role which could play a part in the progression of the neoplastic disease. Further studies will be fundamental in facing pruritus in oncologic patients, since this problem compromise their quality of life worsening an already critic picture.

## Introduction

Pruritus is one of the most disabling symptoms in oncologic patients. It is typical of some diseases like Hodgkin’s lymphoma, leukaemia and cutaneous T-cell lymphoma and biliary cancer [1]. The above-described symptom can be categorized in two subtypes: the first is a direct consequence of the neoplasia in a particular tissue and the second is paraneoplastic. The last type of pruritus was defined by The Special Interest Group of the International Forum on the Study of Itch. They define paraneoplastic itch as a symptom provoked neither by the local presence of tumor cells nor by anti-cancer treatments. It is usually concomitant with the pathology progression or in some cases it can precede a hidden malignancy; once more, it is not caused by the physical presence of the mass and subsides after the recovery [2–4]. In this scenario, inflammatory mediators are fundamental causal agents of skin diseases and neoplastic-associated cutaneous signs. Often, these skin diseases are pruritic [5].

Among these, Cutaneous T cells lymphoma (CTCL) have been associated to augmented levels of pro-inflammatory cytokines and among these, a Th2 cytokine called interleukin 31 (IL-31) appeared particularly involved. Interleukin (IL)-31, a cytokine cloned by Dillon et al. in 2004, is primarily secreted by activated T cells, especially T helper Th2 cells (CD4 + CXCR3- CCR4 + CCR6-cells) [6]. Recently, it was reported that the IL-31 receptor is expressed in the peripheral nerves of mice and humans [7], suggesting that IL-31 secreted by Th2 cells may influence right peripheral nerves, triggering the pruritus linked to atopic dermatitis (AD).

This cytokine, in fact, belong to the IL-6 family, and have been frequently associated to pruritic skin diseases [6]. CD45RO + cutaneous T lymphocytes are responsible for IL-31 production. Its receptor is IL-31R, which is heterodimeric and ubiquitously represented. It has 2 subunits, the IL-31 receptor alpha (IL-31RA) and the oncostatin-M receptor beta (OSMR). These two subunits are expressed by IL-31-activated monocytes [8]. Firstly IL-31 was thought to be secreted only by Th2 and Th1 lymphocytes; recently also mast cells were reported being capable of producing IL-31, as well as monocytes, macrophages, and monocyte-derived dendritic cells and human mast cells. According to these data, innate and adaptive immunity appear to be linked to this interleukin role, in particularly when skin is involved [9–11]. More specifically, intracellular signaling involving the IL-31receptor by IL-31 is facilitated by the Janus kinase (JAK)-signal transducer and activator of transcription (STAT), phosphatidylinositol-3 kinase (PI3K)/AKT, and mitogen-activated protein kinase (MAPK) pathways [12, 13]. The lone phosphorylation of extracellular signal-regulated kinase 1/2 (ERK1/2) is not sufficient to stimulate JAK1/JAK2 and STAT3 [14]. Additionally, it was observed that missense mutations in the OSMR β gene were isolated in patients of familial primary localized cutaneous amyloidosis (FPLCA), hereditary skin disease associated with severe pruritus and deposition of amyloid material in the dermis [15, 16]. OSMRβ is a component of IL-31R and OSM type II receptor. FPLCA keratinocytes were stimulated with OSM and IL-31, the expression levels of phosphorylated STATs, ERK1/2, and AKT were diminished. JAK/STAT, ERK1/2 and PI3K/AKT signaling pathway have anti-apoptotic effect in several tumor cell lines; so apoptosis and accumulation of degenerate keratinous material deposition in the dermis are a possibility in the OSMRβ mutated FPLCA patients. These data indicated that OSM and IL-31 signaling are involved in keratinocyte cell proliferation, differentiation, apoptosis and inflammation [17].

Skin alteration were associated to IL-31 and in every disease the immune system acted a main role: atopic dermatitis (AD), allergic contact dermatitis, prurigo nodularis, and chronic urticaria are some examples. In every pathology itch was present [18, 19].

Circulating IL-31 was directly connected with the severity of itch in a neoplastic disease such as CTCL. In this condition, the mediator was found being released by malignant CD4 + T cells lacking CD26 expression, as well as by cells marked by antibodies against the beta chain variable region (Vβ) of the T cell receptor [20].

**Fig. 1:**
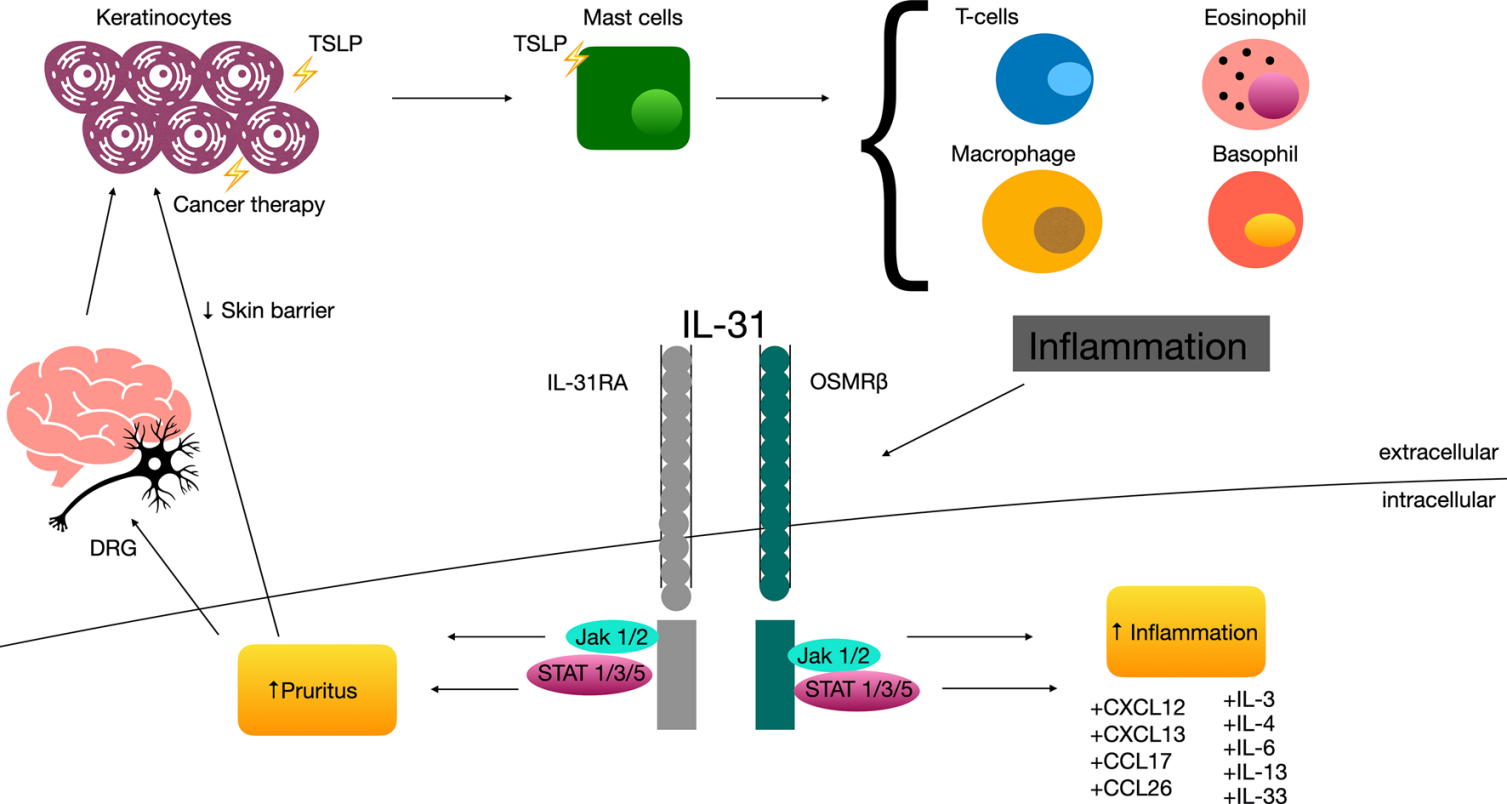
Etiopathogenesis of itch in malignancies linked IL-31

Inflammatory and malignant pruritic skin diseases have an association with Th2 cells and for this reason we aimed to review the interplay between Th2 cells, IL-31, and pruritus in malignancies (Fig. 1)

## Methods

This review was conducted using PubMed database and Google Scholar. A comprehensive search was accomplished employing the following query (date of last search, February 2021): (#1 IL-31; AND #2 Pruritus; OR #3 itch; AND #4 Leukemia, Myeloid, Acute [MH] OR #4 Chronic Myeloid Leukemia; OR # 5 Lymphoma [MH]; OR #6 Cutaneous T cell lymphoma; OR #7 Cancer; OR 8# Malignacies. We included only research articles in English.

In the first phase, titles and abstracts were examined by two researchers, according to precise criteria stated, to regulate this systematic review. Any divergence was decided by a third, independent researcher. We included papers presenting data about the role of IL-31 in the onset of pruritus. In the next phase, full texts of the selected articles were evaluated, and references of included papers were examined to find further works.

The information was then summarized and organized in the present review.

On these websites, we employed as key terms: IL-31, pruritus, itch, cancer, malignancies, Cutaneous T cell lymphoma and leukemia. The selected articles were retrieved in Table 1.

**Table 1:** List of the research articles evaluating IL-31 levels in patients with cancer and pruritus

Authors	Type of diseases	IL-31 levels	Correlation between IL-31 and itch	Valuation of other cytokines	References
Nattkemper et al.	CTCL	↑	Yes	Not evaluated	[24]
Ohmatsu et al.	CTCL	↑	Yes	slL-2R	[25]
Singer et al.	CTCL	↑	Yes	Not evaluated	[20]
Malek et al.	CTCL	↓	No	Not evaluated	[26]
Cedeno-Laurent et al.	CTCL	↑	Yes	Not evaluated	[21]
Ferretti et al.	FL	↑	Not evaluated	Not evaluated	[11]
Musolino et al.	CML	↑	Yes	IL-33	[19]
Ferretti et al.	HL	↑	No	TSLP	[30]

*HL* Hodgkin lymphoma, *CTCL* cutaneous T cells lymphoma, *FL* follicular lymphoma, *TSLP* thymic stromal lymphopoietin, *sIL-2R* soluble interleukin-2 receptor, *IL-33* interleukin-33, *CML* chronic myeloid leukemia

## Results

### Cutaneous T cells lymphoma

Primary cutaneous T-cell lymphomas (CTCLs) account for approximately two-thirds of all primary cutaneous lymphomas [21], with mycosis fungoides (MF) and Sézary syndrome (SS) being the most common subtypes. Both MF and SS are characterized by a monoclonal proliferation of mature T helper lymphocytes in the skin. Tumor cells in MF are classically CD3^+^CD4^+^CD8^−^, with frequent loss of CD7 [22]. Pruritus is typical of most advanced stages of CTCL; often the symptom compromise patients life quality [21]. Th2 cytokines are involved in CTCL pathogenesis of pruritus and include IL-4, IL-5 and IL-13, together with eosinophilia and IgE [23].

Nattkemper et al. [24] studied for the first time the expression levels and patterns of IL-31 and both of its receptors within the skin of CTCL subjects with pruritus. According to their data IL-31 was positively linked to itch level. However, IL-31 did not match with disease stage. Probably, IL-31 is not fundamental for the pathogenesis of CTCL. One study bias was the low number of early stages CTCL subjects; moreover, CTCL patients without pruritus were totally missing. IL-31RA and oncostatin-M receptor beta (OSMRβ) were highly expressed too. IL-31 was significantly increased in skin and in the lymphocytic infiltrate, but on the other side, its receptors were augmented only in the epidermis.

Also Ohmatsu et al. [25] demonstrated augmented circulating IL-31 in CTCL; but also they demonstrated the presence of several cytokines (i.e. IL-4 and IL-6) and chemokines (i.e. CXCL12, CXCL13, CCL17 and CCL26) fundamental in CTCL which might be modulated by IL-31. In this case CTCL was as severe as IL-31 was higher. Serum sIL-2R and lactate dehydrogenase (LDH) were also correlated to the severity.

In another report, 40 patients had a statistically significant variation in IL-31 levels between patients affected by itch and non-pruritic subjects. According to the authors CD4 + CD26- malignant cells specifically released IL-31. Finally the withdrawal of the itching sensation was correlated to lower levels of serum IL-31 [20].

The study by Malek et al. reached partially different conclusions. IL-31 was higher in advanced stages (IV) of CTCL the in earlier (IA). There was a low correlation between serum IL-31 concentration and pruritus in the whole patients. These contrasting data versus other studies, were probably due to the different severity of the subjects considered [26].

Blocking IL-31 improved itch in patients with advanced stages of CTCL. Dexamethasone and vorinostat reduced IL-31 levels and their producing cells with no effects on other malignant T cells highlighting the interleukin importance in pruritus pathogenesis but not in the disease [12].

### Follicular lymphoma

Follicular lymphoma (FL) is a systemic neoplasia of the lymphoid tissue characterized by germinal center (GC)-derived B cell malignancy and differentiation. It constitute about the 5% of all hematologic malignancies and about the 20–25% of the new diagnosed non-Hodgkin lymphoma in the industrialized countries [27].

Ferretti et al. reported the first data about the IL-31/IL-31R complex in B-cell lymphomas [11]. They analysed this mature B-cell malignancy, which is the second most frequent B-cell lymphoma after large B-cell lymphoma. They noticed that IL-31 and its receptor have a paracrine/autocrine role in favouring FL progression. Both CD4 T cells and CD68 macrophages were found capable of producing IL-31.

### Hodgkin lymphoma

Hodgkin Lymphoma (HL) is a B cell-malignancy with low ratio of neoplastic mono-nucleated Hodgkin and multi-nucleated Reed-Stenberg (H/RS) cells in the invaded lymph nodes [28]. These cells are influenced by the interplay between tumour cells and reactive cells accumulating in HL-involved tissues. These non‐malignant cells, recruited and/or induced to proliferate by tumour cells, release soluble or membrane‐molecules with a role in malignant cells growth and survival [29].

Surface and cytoplasmic IL-31 and thymic stromal lymphopoietin (TSLP) and their receptors were studied in H/RS cells. Lymph node microenvironment with immune and malignant cells coexisting with these mediators give an idea of the complexity of this interaction. In this scenario, the authors did not find augmented levels of IL-31 in the overall patients group respect to healthy subjects. However, both levels of the interleukin and of the pruritogenic cytokine TSLP appeared to be intimately linked. The authors found no association between itch and IL-31 and between disease stage and IL-31 in HL subjects. However, in patients with a worst prognosis they found higher levels of sIL-31 and sTSLP compared to those with a better possible outcome. This difference suggested a prognostic potential of these parameters [30].

### Chronic myeloid leukemia

Imatinib mesylate belong to the family of the tyrosine kinase inhibitor (TKi); it’s commonly used for the treatment of chronic myeloid leukemia (CML). Skin disorders were considered a direct effect of the drug rather than an adverse reaction [31, 32]. We performed a study on a CML patient with an intense pruritus developed after Imatinib mesylate administration. The patient had elevated plasma levels of both IL-31 and IL-33. We speculated that TKi could directly provoke keratinocyte disruption releasing IL-33, which in turn activated an inflammatory cascade responsible for involving IL-31 and causing skin damages [19].

## Discussion

### Interleukin-31, pruritus and the CTCL model

Evaluating IL-31 role in pruritus pathogenesis have to necessarily consider both the plasmatic interleukin and its relative receptors: IL-31 receptor A and the oncostatin M receptor [33]. IL-31 receptor A is mainly represented on the sensory dorsal root ganglion; on the other side, oncostatin M receptor was linked to pruritus due to its connection with cutaneous amyloidosis. Also, IL-31 mRNA had a key role in pruritus since it high levels in pathologies intimately linked to it: atopic dermatitis, allergic contact dermatitis and prurigo nodularis [18]. Moreover, the interleukin is also correlated with the severity of the diseases [34]. IL-31 role in CTCL was confirmed by the above cited studies, where the mediator was often correlated with advanced stages of the disease in symptomatic patients [20, 26]. Contrasting data about earlier and latter stages may be due to the Th response; in the first phase of the disease in fact, there is a predominance of Th1 phenotype. It is known that IL-31 is produced mainly by Th2 lymphocytes and this is typical of advanced stages of CTCL [10]. Once more, high blood levels of IL-31 were linked to pruritus intensity and serum and tissue expression of the chemokine CCL18 [35]. IL-31 was also found to be augmented in advanced stages of mastocytosis and the immunohistochemical analyses of bone marrow confirmed that tryptase-positive mast cells as were the major source of the interleukin [36]; in addition some IL-31 gene polymorphisms were reported to amplify both mast cells involvement and pruritus [37]. Continuing talking about Th2 cytokines, Stott et al. [35] demonstrated that IL-31 expression is modulated by IL-4. According to their data, the blocking of IL-4 from a Th2 environment diminished the levels of IL-31. Several authors also showed the induction of IL-31 by IL-33 [19, 35]. Moreover, it is thought that IL-31 can induce keratinocytes and infiltrating cells to release additional mediators involved in pruritus. Furthermore, IL-31 has also been shown to cause the release of pro-inflammatory cytokines from eosinophils, monocytes, and macrophages [38]. The IL-31/33 axis role in pruritus in malignancies should be deeply investigated. Finally, patients affected by CTCL with skin disorders were demonstrated to express Th2 cytokines in their lesions: IL-4, 5, 6, 9, 10,13, 31 (and its receptors) and 33. Th2 expression augment in consideration of disease stage and this could in part explain pruritus progression [39].

These results give birth to the need of more studies about IL-31 role in malignant itch. For these conflicting data, new research need to investigate the role of IL-31 in pruritus associated to malignancies.

### The neuro-immune hypothesis

IL-31 seems to induce pruritus by directly stimulating IL-31RA on TRPV11/ TRPA11 sensory nerves in the skin [7]. IL-31 and that the number and activation of Th2 cells, were augmented in human and animal models of AD suggesting a neuroimmune cycle involving Th2 cells, and IL-31RA on sensory nerves that seems applicable on the neoplastic model. On the other hand, the other IL-31 receptor, OSMRb was not elevated suggesting a minor importance in provoking itch. Some researchers proposed the hypothesis that IL-31RA might be considered a functional neural cytokine receptor [7]. One of the most used drugs in order to control pruritus, also in oncology, are anti-histamines but this speculation emphasizes that not only mast cells through histamine or tryptase [40] but also T cells through IL-31 could straight stimulate sensory nerves [7].

The nervous system involvement was endorsed by dorsal root ganglia (DRG) expression of IL-31 and IL-31RA after the cutaneous administration of the interleukin capable of provoking pruritus [41].

## Conclusions

Itch is often associated to cancer. It could be mainly associated to 3 situations. First, it could represent the first signal of the tumor presence and constitute a paraneoplastic syndrome. Its causative factor is the systemic release of oncologic mediators. Second, pruritus could be generated by the physical presence of the malignant mass. Third, itch could be a consequence of anti-neoplastic drugs. We described a potential fourth actor in the scenario constituted by an oncologic patient: an interleukin capable of provoking pruritus without being part of the first three pathways described above.

The data collected in this review showed that high IL-31 levels and its receptors are correlated to skin pruritus in malignant disease. The most studied model was CTCL, where the pathogenetic mechanism seems to be emblematic. The researches collected together with other reports were not able to clarify if interleukin 31 is connected to the itch, to the cancer or to both [42]. These data are encouraging in respect to a potential anti-pruritic treatment based on reducing IL-31 or its receptor IL-31RA. Nemolizumab, as a monoclonal antibody against this interleukin, demonstrated its efficacy in controlling pruritus in other diseases and offers a hope for oncologic patients [43–46]. However, CTCL is not the only malignant disease existing. In fact, one of the messages of this review is to stimulate researchers in linking itch in various type of cancer and testing the role of the “skin neuroimmune cytokine” IL-31. Results will be fundamental in facing pruritus in oncologic patients, since this problem compromise their quality of life worsening an already critic picture.

## Data Availability

Not applicable.
